# Inhaled Medications in Chronic Respiratory Diseases: Analysis of Real-Life Use in Puglia (Apulia), Italy

**DOI:** 10.3390/jcm12062436

**Published:** 2023-03-22

**Authors:** Giulia Scioscia, Pasquale Tondo, Maria Grazia Cagnazzo, Anela Hoxhallari, Francesco Satriano, Giulio Rollo, Donato Cinquepalmi, Antonio Grieco, Maria Pia Foschino Barbaro, Donato Lacedonia

**Affiliations:** 1 Department of Medical and Surgical Sciences, University of Foggia, 71122 Foggia, Italy; 2 Department of Specialistic Medicine, Institute of Pulmonary Medicine, “Policlinico Foggia” University Hospital, 71122 Foggia, Italy; 3 Pulmonology Unit, “Vito Fazzi” Hospital, 73100 Lecce, Italy; 4 Local Health Authority of Lecce (ASL/LE), 73100 Lecce, Italy; 5 Health Management, Hospital Institution Pia Fondazione di Culto e Religione “Card. G. Panico”, 73039 Tricase, Italy; 6 Medical Department, GSK, 37135 Verona, Italy; 7 Department of Innovation Engineering, University of Salento, 73100 Lecce, Italy

**Keywords:** asthma, COPD, chronic respiratory diseases, respiratory failure, therapy

## Abstract

Background: Chronic respiratory diseases (CRDs) are common diseases with a heterogeneous distribution worldwide. Due to their impact on disability, weight assistance and pharmaceutical spending, they represent an important global burden for national health systems. However, few studies have investigated the use and consumption of inhaled drugs in real life in patients with CRDs. Objective: This study aimed to investigate the real-life consumption of health care resources of main CRDs through an analysis of the administrative databases of the local health authority (ASL) in the Puglia region (Italy). Methods: The present study is an observational study that longitudinally reviewed the administrative and health databases associated with patients’ consumption of health resources between 2017 and 2018. Results: The first important finding is a marked underestimation of the true incidence of CRDs despite the search for disease-specific exemption codes. Another important result is that the real-life consumption of inhaled drugs among these patients is well below the minimum acceptable values for adherence. The most commonly used inhaled drugs, for which at least one pack was withdrawn, were inhaled steroids (61.6%), followed by the ICS/LABA combination (43.7%) and LABAs (32.4%). However, less than one-third of patients (31%) withdrew at least three packages of ICS or ICS/LABA during the year, while the percentage was reduced to less than 15% for other combinations. Another alarming finding is that only 8.4% of patients taking CRDs drugs reported at least one spirometry during the study period. Conclusions: The wide availability of computerized systems may be an important tool for increasing therapeutic adherence and optimizing the resources of health systems in the diagnosis, treatment and management of patients with CRDs.

## 1. Introduction

Chronic diseases are the world’s major public health problem as they are the leading causes of morbidity, disability and mortality and have a high social and economic impact [1]. In particular, chronic respiratory diseases (CRDs) represent an outstanding part of chronic diseases due to their relevance in terms of epidemiology, severity, disability, mortality and burden of care [2].

When analyzing the situation in Italy, CRDs are the third leading cause of death after cardiovascular and malignant diseases. In addition, given the aging population, their prevalence is expected to rise in the next decades [3].

The Italian Decree of the President of the Council and of the Ministers (DPCM) of 12 January 2017 has identified three main chronic respiratory diseases: asthma, chronic obstructive pulmonary disease (COPD) and chronic respiratory failure (CRF) [4]. To take care of patients with chronic diseases, the State-Regions Conference approved the National Chronicity Plan (NPC). The aim of the NCP is to protect people with chronic diseases and to reduce their burden on the individual, their family and the social context by improving quality of life. In addition, it encourages the implementation of telemedicine service centers that integrate devices operating in the territory.

Additionally, to make the treatment organization more efficient and appropriate, health systems in Italy have implemented the Diagnostic and Therapeutic Care Pathways (PDTA) [5].

However, given the heterogeneity of regional health care systems, it is very difficult to assess the real impact of chronic diseases on a territory. As a result, there are no criteria for measuring the real effects of the diagnosis, treatment and care programs of regional or local health authorities, nor is there a systematic survey of clinical outcome indicators to check the adequacy of resource use.

For this reason, the National Agency for Regional Health Services (AGENAS) promoted the MATRICE project (“Integration of information content for the territorial management of patients with complex or chronic diseases”) [6] to monitor the PDTA. This project represents a model for integrating information on all services (hospital, diagnostic, specialist, therapeutic, etc.) for specific chronic diseases obtained by the New Health Information System through a computer application.

Therefore, the present study was designed to assess the incidence of the three main chronic respiratory diseases in the Puglia region and their relative consumption of health care resources using the algorithms established in the PUGLIA CARE 3.0 project [7]. This model is based on taking care of a chronic patient through the provision of a rational care plan that adheres to national and local guidelines, promoting adherence to follow-up by the chronic patient and making care services more easily available in the territory of residence by offering an efficient level of care to the entire population.

In practice, in the PUGLIA CARE 3.0 project, several professional profiles are involved: a general practitioner who will have to frame the patient’s needs on a clinical and social level and design an individual pathway on the basis of national and international guidelines; a practice nurse who will assume the functions of case manager (assistance and liaison between the various figures involved in the care pathway); and a practice assistant who, in addition to taking care of the computer management of the process, will assist the nurse in supporting the management of the patient’s administrative practices (e.g., reservations for specialist services).

## 2. Methods and Data Analysis

This study was conducted based on data collected by the local health authority (ASL) in Lecce between 2017 and 2018. The administrative and health province databases were reviewed longitudinally, with the databases being associated with each patient and assisting in managing their contacts and consumption of health resources.

The administrative flows used were the following: register of patients; disease-specific exemptions; hospital discharge cards; outpatient specialist services; approved drugs for specific diseases; home care assistance; granting of disease-specific devices/assistance provided by the nomenclator; long-term liquid or gaseous oxygen treatment programs; residential and semi-residential care; and administration of influenza and anti-pneumococcal vaccines.

The following data were included in the analysis:

- Disease-specific exemptions (see Section 2.1). Ticket-exempt registers for asthma, COPD and CRF contain information regarding tax code, GP code, exemption code, beginning of exemption, end of exemption, district code, sex, date of birth and residence (Table 1).
- Hospital discharge card (SDO) is a nationwide instrument for collecting information on every patient discharged from all public and private inpatient institutions. The SDO has a unique code according to the diagnosis that is generated upon hospital admissions made in the 2017–2018 biennium and contains information on the following: social security number, district code, gender, date of birth, residence, card number, start date of hospitalization, end date of hospitalization, days of hospitalization, principal diagnosis and secondary diagnosis.
- Specialist services during 2017–2018 contain information regarding tax code, district code, gender, date of birth, residence, prescription ID, description, quantity, amount and month.
- Pharmaceutical prescriptions between 2017 and 2018 contain information regarding tax code, district code, sex, date of birth, residence, ticket exemption, Anatomical Therapeutic Chemical (ATC) classification system, drug identification code, amount, number of packages and month of dispatch (Table 2).

However, records of drugs purchased by the ASL from a manufacturing company and dispensed to patients for home administration, either through healthcare companies (direct distribution) or through specific agreements with local public and private pharmacies, as defined in the Ministry of Health Decree of 31 July 2007, were excluded [8].

### 2.1. Exemption Code

The Italian National Health Service (NHS) requires citizens to participate in healthcare spending through a ticket, which is the amount people pay at the front desk, before a clinical exam or visit, or at the pharmacy, when they pick up a prescription drug from the Regional Health Service. Paying the ticket allows all citizens to receive care under the Essential Levels of Care (LEA), i.e., services and benefits free of charge. At the same time, the ticket avoids the provision of unnecessary health services by the NHS. Citizens may be exempted from the payment of the ticket in some cases:

- Income exemption;
- Exemption for chronic diseases;
- Exemption for rare diseases;
- Exemption for disability;
- Exemption for blindness and civil deafness.

Each chronic disease or rare disease is marked with a specific exemption identification code. There are 64 chronic and disabling diseases/conditions that are entitled to exemption from a ticket, according to the provisions of the new LEA approved in the Decree of the President of the Council of Ministers (D.P.C.M 12 January 2017) “Definition and updating of the Essential Levels of Care”.

The right to exemption is recognized by the ASL of residence based on the certification of the disease released by a specialist on the appropriate form. It is possible to apply for exemption for more than one disease.

### 2.2. Data Interpretation

The data presented in the study were expressed as mean ± standard deviation in the case of continuous variables or as number and/or percentage in the case of categorical variables. The longitudinal trends of the data were observed for a better understanding of long-term therapeutic adherence. All graphs were constructed using Excel (Microsoft Corporation, Redmond, WA, USA).

## 3. Results

### 3.1. Population Characteristics and Sample Stratification

The information flows of about one million patients in the Lecce ASL were examined.

Only 167,226 patients were identified as being affected by respiratory diseases, of which 63,716 (8% of overall examined population) with chronic obstructive respiratory diseases (asthma, COPD and CRF). The cohort was predominantly male (52%), with a mean age of 54.5 years.

When stratifying the sample by the exemption codes, it emerged that 83% of the sample (52,854 subjects, with an average age of 55.2 years) had no specific exemption code for CRDs; 11% (average age of 39.3 years) had an exemption code for asthma; 0.4% (average age of 67.9 years) had an exemption code for COPD; and 5.6% (average age of 73.9 years) had an exemption code for CRF. By searching the sample of CRDs for exemption codes by economic condition, age or disability, it emerged that 72.2% (46,023 subjects, with an average age of 59.8 years) already had another ticket exemption. Specifically, 68.4% had an exemption for economic condition and age, and 31.6% (average age of 66.7 years) had an exemption for partial or total disability (Table 3).

The flow distribution of the subjects with CRDs, calculated according to their age, is characterized by two peaks: the first peak falls around 16 years of age and the second around 70 years of age. As it is to be expected, asthma prevails in the first peak and CRF in the second.

**Figure 1:**
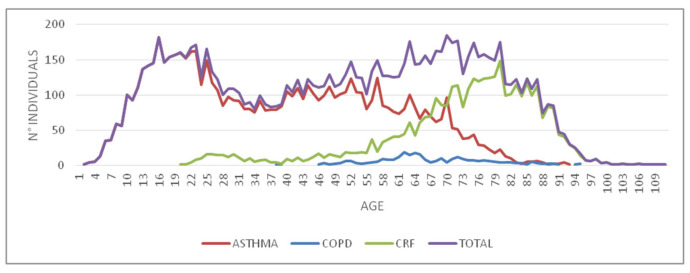
Age distribution of individuals with a chronic respiratory disease (CRD) according to the Local Health Authority (ASL) of Lecce between 2017 and 2018.

In contrast, the incidence of asthma alone shows two peaks: the first at 22 years of age and the second at 58. In the case of COPD and CRF, the highest number of subjects is concentrated at 64 and 80 years of age, respectively (Figure 1).

### 3.2. Consumption of Inhaled Drugs

During the two years of observation, 793,985 prescriptions were examined. A total of 760,915 prescriptions were for drugs with the R03 code (respiratory drugs used for obstructive diseases according to the Anatomical Therapeutic Chemical classification system) (Table 4). The total number of patients who benefited from these prescriptions was 162,174, with a total expenditure of 21,275,298.89. The number of R03 drugs dispensed to subjects with CRD was a total of 611,103 packages (average of 10.5 packages/subject). Of these, 10% of the consumption was attributable to patients who withdrew more than 50 packs in the two years.

A total of 64,419 (10.5%) packages were dispensed to subjects with an asthma exemption code (mean 11.9 packs/subject); 2896 (0.5%) to subjects with a COPD exemption code (mean 13.9 packs/subject); and 54,746 (9%) to subjects with a CRF exemption code (mean 20.5 packs/subject). The remaining 489,042 packs (80%) were dispensed to subjects with another exemption code or without an exemption code for CRD (mean 9.8 packs/subject).

The majority of respiratory drug consumption was attributable to patients who were exempted for other conditions compared to those who did not have exemptions.

**Figure 2:**
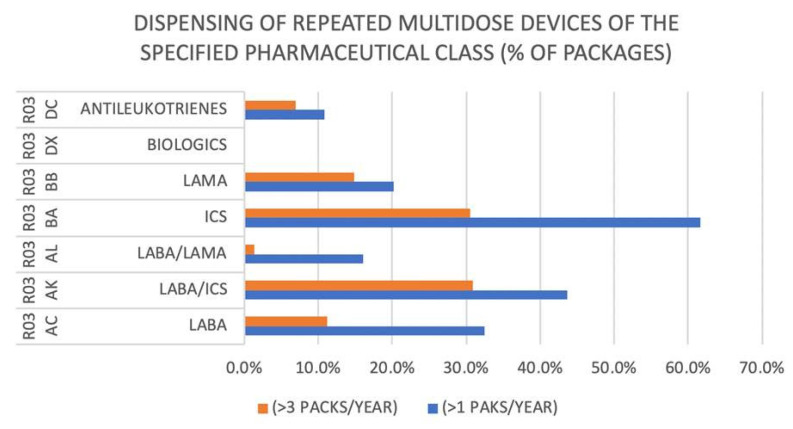
Evaluation of adherence to several respiratory medications (Anatomical Therapeutic Chemical classification code R03).

When analyzing inhaled drugs for CRDs, the most widely used were inhaled steroids (ICS, 61.6%), followed by inhaled corticosteroid (ICS)/long-acting β2 adrenergic (LABA) combination (43.7%) and LABA alone (32.4%). Among the least-dispensed inhaled drugs were long-acting muscarinic antagonists (LAMA) (20.2%) and the LABA/LAMA combination (16.6%). These percentages were considerably reduced when considering those subjects who withdrew at least three packages. In detail, when analyzing repeated dispensations by single pharmaceutical class, 19,470 patients (31%) used at least three packages of ICS, 19,668 (31%) used at least three packages of ICS/LABA, 94.70 (15%) used at least three packages of LAMA, and 7173 (11%) used at least three packages of LABA (Figure 2). Most subjects with CRDs (44%) consumed between 4 and 12 packages/year of R03 medications (single and multidose). One third of the subjects used less than 3 packages per year, while 24% took more than 12 packages per year, and of these, almost half took more than 24 packages per year. In the observational period, only 8.3% used the same drug, while the remaining changed drug and/or device, often also changing the class of inhaled drug.

Finally, Table 5 shows the combinations of several drugs (and devices) in those already receiving at least three packs of a single drug. The data do not include any changes during the year.

From Table 5, it can be gathered that the number of subjects taking an open-label triple therapy (ICS/LABA + LAMA) amounted to 7973 patients with one package and 5069 patients with three packages. In addition, a progressive decline in adherence for open-label triple therapy (ICS/LABA + LAMA) was observed over one year, ranging from 43% in the first 6 months to 24% after 12 months (Figure 2).

The consumption of medicines used as required (salbutamol and ipratropium) was also analyzed: consumption peaked in the 2–5 age group (2.03 packs/year on average) in children, and consumption peaked between the ages of 69 and 83 (2.62 packs/year) in adults.

In addition, an analysis of all specialist services provided for the sample with CRD showed that only 17% had specialist pulmonology services. Comparing the data on drug consumption with the data on services provided, it emerged that 32.9% of subjects with a COPD exemption had performed at least one spirometry test (simple or global) in the two years examined, while the rest had not performed any spirometry tests (at least in the observation period). This finding was significantly lower among subjects with CRF (13%) and among those with asthma, where only 7.5% performed at least one spirometry per year, as well as in patients who had no disease exemption (7.2%). Overall, only 8.4% of patients taking medication for CRDs performed at least one spirometry test in the two observation years.

## 4. Discussion

CRDs remain a leading cause of death and disability worldwide [9,10,11,12]. Likewise, healthcare costs for these diseases are a growing burden on the economies of all nations. Despite the extensive health and economic consequences, there is a lack of comprehensive published data on the prevalence and distribution of CRDs on a global scale [2]. For this reason, the Global Alliance Against Chronic Respiratory Diseases (GARD), a voluntary alliance at the national and international levels, was set up since 2004; this alliance is made up of organizations, institutions and agencies working to make CRDs a public health priority in all countries. It also aims to ensure that governments, the media, citizens, patients, health professionals and all stakeholders are aware of the problem. The World Health Organization (WHO) has also emphasized that Italy needs to make further efforts for the management of chronic patients through the implementation of the National Plan for Chronicity (NPC). It consists of an elaboration of diagnostic and therapeutic pathways (PDTA) that have to be adopted at both the territorial and hospital levels for organizing the diagnosis, treatment and management of chronic diseases (including CRDs) in order to create integrated hospital–territory pathways that guarantee equal access and services to affected patients over national territories [5]. At the same time, it is necessary to create multi-specialist hospital networks, which promote integration with the local area and ensure continuity of care. However, all these actions cannot be implemented adequately if they are not supported by continuous data monitoring of the actual prevalence and burden of CRDs.

Our study represents an analysis of real-life data relating to a large area of the Apulia region using simple indicators (disease exemption codes for asthma, COPD and CRF) to identify the actual impact of each disease on the territory. The results showed that only 11% of patients had an exemption code for asthma, 0.4% for COPD and 5.6% for CRF. The remainder of patients (73% of the subjects) with CRDs applied for co-payment exemption for other reasons (income, disability and other conditions). As a result, it was not possible to correctly stratify the patients according to their respiratory diseases [13]. Specifically, the analysis highlighted that research by disease-specific exemption codes leads to a significant underestimation of the actual incidence of CRDs. Similarly, other studies showed that the prevalence of COPD and asthma can be estimated from disease-specific drug codes, hospital discharges and, when available, co-payment exemptions only with high systematic uncertainty [14,15,16]. However, it is noteworthy that the exemption code for COPD (code 057 in Italy) has only recently been introduced, and this may partly justify its low diffusion on the territory.

In addition, we calculated an indirect estimate of adherence to these drugs through the consumption data; the finding showed that subjects treated with ICS, and even more so with ICS/LABA, presented a higher therapeutic adherence (consumption of >3 packages/year at least) than the other drug classes. In this regard, we should consider that measuring medication adherence is extremely complex because inhaler consumption data do not indicate whether the medication is being taken correctly [17,18]. Indeed, a recent study investigating pharmacy claim data to assess refills of ICS/LABA versus records of inhaler sensors to monitor daily use patterns of the same medication revealed that claim-based measures of adherence were higher than sensor-based measures [19].

Additionally, the data on the variation of therapy over time are very interesting. In fact, in the two years covered by our study, more than 90 percent of subjects changed their therapy by changing the device and very often changing the class of inhaled drugs as well. The change in therapy could be due to a variety of reasons: lack of effectiveness of the therapy previously taken, step-up or step-down changes in therapy depending on the course of the disease, difficulties with the device in use, the introduction of new drugs in the market, etc. This evidence highlights the wide variability of therapies taken by patients with CRDs, which may not always have a positive effect on the disease and on treatment adherence.

From this perspective, it is noted that patients with long-term and polypharmacy therapies tend to have a poor adherence to treatment. In fact, very few patients consumed 10–12 packages of any inhaled drug per year, which, as we know, requires therapeutic continuity to be effective. To confirm this, a recent three-year real-life study on inhaled medications used in the treatment of COPD showed that treatments requiring a once/day dose had a significantly better adherence compared to those with multiple daily doses and that adherence to therapy progressively decreased over the years [20]. To strengthen this evidence, our study pointed out that only a quarter of the patients continued an open-label triple therapy (ICS/LABA + LAMA) for about 12 months after the first prescription. Consequently, this result contrasts with the fact that patients on a triple therapy should have higher adherence because they are more severe and symptomatic.

Additionally, the low number of spirometric examinations performed by patients with CRDs is a discouraging result for the management of such patients. Indeed, the use of spirometry is essential for an accurate diagnosis of respiratory disease and for monitoring long-term progression and therapeutic efficacy. However, this examination is underused in both primary and specialist care [21]. Different studies have highlighted how airway obstruction remains seriously under-diagnosed and, therefore, untreated in hospitalized patients, not only at the time of admission but also at the time of discharge [22,23]. Several factors may contribute to this result, namely lack of familiarity with the use of a spirometer, lack of confidence in interpreting the reported parameters, lack of availability of equipment, and lack of encouragement to perform the examination [24]. This leads to a high rate of misdiagnosis in patients with suspected chronic airway inflammatory diseases [25]. Thus, more explicit promotion is needed regarding the role of spirometry in the diagnosis and management of CRDs. Recently, the introduction of the Note 99 by the Italian Medicines Agency (AIFA) [26], which requires spirometry to be performed for drug prescriptions, is expected to lead to a progressive increase in the number of spirometry examinations in patients with COPD and, consequently, to a better management of this disease.

Therefore, monitoring therapy use through a network of hospital, territorial and local facilities on a large scale could improve chronic disease management through early detection of problems related to patient adherence or mismanagement. As a consequence, better patient management would result in a better quality of life, as well as a reduction in mortality rates and healthcare expenditure related to CRDs.

## 5. Limitations of the Study

This study has many limitations. The most important one is that the data come from a limited area in southern Italy. In addition, the incidence of chronic respiratory diseases is an estimated figure in the absence of an accurate computer system, which is just recently being implemented somewhat throughout Italy. Another limitation is the lack of data concerning inhaler type. Nevertheless, the issues raised in this study affect not only Italy but may also apply to the whole world, such as adherence to inhaled therapy, underdiagnosis of chronic respiratory diseases, and limited number of spirometry and specialized pulmonary services.

## 6. Conclusions

Assessing the burden of CRDs by means of valid and up-to-date estimates of their prevalence may help guide a better management of healthcare resources.

Our study, therefore, attempted to evaluate the management of these diseases by means of exemption codes, demonstrating the inherent limitations of this procedure related to the overlapping of different exemptions that may not allow for reliable estimates.

However, this procedure also showed potential because it allowed us to estimate the number of patients with CRDs based on the purchase of respiratory drug packages and, consequently, the rate of adherence to treatment by evaluating sales over time; in addition, we found out whether the management of patients with CRDs, e.g., the performance of spirometric tests in the expected numbers and times, is performed correctly.

Therefore, given the computerization of the healthcare system, improving these procedures for monitoring patient flows could have useful implications for the development of new health management and organizational systems for chronic diseases.

## Figures and Tables

**Table 1:** Identification codes (exemption codes, SDO/DRG codes and ATC codes) for subjects with chronic respiratory diseases according to PUGLIA CARE 3.0.

Description	Flows	Code
Exemption code		
COPD		057
Asthma		007
CRF		024
SDO/DRG (Admission/Health Service)		
Chronic bronchitis		491
Emphysema		492
Asthma		493
Bronchiectasis		494
Unclassified chronic airway obstruction		496
Acute respiratory failure		518.81
Chronic respiratory failure		518.83
Chronic and acute respiratory failure		518.84
ATC codes (drugs)		
Drugs for obstructive airway diseases		R03
Drugs for heart disease		C01–09
Drugs for diabetes		A10

Abbreviations: A10 = list of hypoglycemic drugs; ATC = Anatomical Therapeutic Chemical classification; C01–09 = list of cardiovascular disease drugs; COPD = chronic obstructive pulmonary disease; CRF = chronic respiratory failure; DRG = diagnosis-related groups; R03 = list of respiratory disease drugs; SDO = hospital discharge cards.

**Table 2:** Anatomical Therapeutic Chemical (ATC) classification system of respiratory drugs reported in the study.

Description	ATC Code
Glucocorticosteroids and inhaled corticosteroids (ICS)	RO3AB
Long-acting muscarinic antagonists (LAMA)	R03BB
Selective long-acting beta 2 adrenergic receptor agonists (LABA)	RO3AC
Long-acting beta 2 adrenergic combined with corticosteroids (ICS/LABA)	RO3AK
Long-acting beta 2 adrenergic combined with long-acting muscarinic antagonists (LABA/LAMA)	R03AL
Leukotriene receptor antagonists (Leukotrienes)	R03DC
Other biological drugs for obstructive respiratory diseases for systemic use (Biological drugs)	R03DX

Notes: Salbutamol belonging to ATC class R03AC02 and salbutamol and ipatropium bromide belonging to ATC class R03ACL2 were also included in the study.

**Table 3:** Stratification of the sample with chronic respiratory diseases (CRDs) and sub-classes of asthma, chronic obstructive pulmonary disease (COPD) and chronic respiratory failure (CRF) by income and disability exemption code.

Variables	Code	Total	Asthma	COPD	CRF
Sample		46,023	4860	156	2579
Sex			F: 2550; M: 2310	F: 48; M: 108	F: 1048; M: 1531
Age		59.8	41.3	69.6	75.4
INCOME-BASED EXEMPTION	E03	3081 (7%)	63 (1%)	8 (5%)	305 (12%)
	E04	1108 (2%)	24 (0%)	5 (3%)	85 (3%)
	E94	19,051 (41%)	935 (19%)	66 (42%)	502 (19%)
	E95	3296 (7%)	56 (1%)	11 (7%)	146 (6%)
	E96	4965 (11%)	221 (5%)	15 (10%)	128 (5%)
DISABILITY EXEMPTION	TOT 01	7061 (15%)	68 (1%)	17 (11%)	772 (30%)
	TOT 02	62 (0%)	3 (0%)		2 (0%)
	TOT 04	7355 (16%)	3490 (72%)	34 (22%)	688 (26%)
	TOT 05	20 (0%)			1 (0%)
	TOT 07	1 (0%)			
	TOT 08	10 (0%)			
	TOT 09	9 (0%)			
	TOT 10	4 (0%)			

All data are expressed as number and (percentage). Notes: E03 and E04: income-based exemption; E94, E95 and E96: code for full or partial exemption of the cost of drugs; TOT 01: 100% civil invalids or work-related invalids (from 80 to 100%); TOT 02: war invalids; TOT 04 and TOT 05: full exemption for pathology: TOT 07: Abruzzo Region residents affected by the seismic events of 6 April 2009, temporarily hosted in Puglia; TOT 08: foreign child (EU and non-EU) awaiting adoption or subject to a guardianship measure (family foster care, group home, etc.); TOT 09: asylum applicant/international protection; TOT 10: non-EU foreign nationals, not in good standing with the norms regarding entry and residence.

**Table 4:** The consumption of respiratory drugs (packages) in patients with specific exemption codes, those without exemption code, and those with other exemption codes.

	Consumption of Respiratory Drugs (R03)		
	Patients n. (%)	Packages n. (%)	Mean Packages
Asthma	5406 (9.3)	64,419 (10.5)	11.9
COPD	208 (0.4)	2896 (0.5)	13.9
CRF	2674 (4.6)	54,746 (9)	20.5
None	50,135 (85.8)	489,042 (80)	9.8
Total	58,423 (100)	611,103 (100)	10.5

**Table 5:** The percentages represent the ratio of number of patients taking at least 3 packages of two drugs to the total number of patients.

Medications	LABA	LAMA	ICS	ICS/LABA	LABA/LAMA	Leukotrienes	Biologics
LABA		20.8%	36.8%	22.3%	1.5%	9.3%	0.1%
LAMA	20.8%		21.5%	53.5%	2.4%	4%	0.3%
ICS	36.8%	21.5%		16%	4.2%	5.6%	0.1%
ICS/LABA	22.3%	53.5%	16%		1.5%	10.9%	0.2%
LABA/LAMA	1.5%	2.4%	4.2%	1.5%		4.8%	0.2%
Leukotrienes	9.3%	4%	5.6%	10.9%	4.8%		0%
Biologics	0.1%	0.3%	0.1%	0.2%	0.2%	0%	

Abbreviations: Biologics = biological drugs for obstructive respiratory diseases; ICS = inhaled corticosteroids; ICS/LABA = long-acting β2 adrenergic combined with inhaled corticosteroids; LABA = selective long-acting β2 adrenergic receptor agonists; LABA/LAMA = long-acting β2 adrenergic combined with long-acting muscarinic antagonists; LAMA = long-acting muscarinic antagonists; Leukotrienes = leukotriene receptor antagonists.

## Data Availability

The data that support the findings of this study are available from the corresponding author upon reasonable request.
